# Developing and Applying Geographical Synthetic Estimates of Health Literacy in GP Clinical Systems

**DOI:** 10.3390/ijerph15081709

**Published:** 2018-08-10

**Authors:** Gill Rowlands, David Whitney, Graham Moon

**Affiliations:** 1 Institute of Health and Society, Newcastle University, Newcastle-upon-Tyne NE2 4BN, UK; 2 Division of Health and Social Care Research, King’s College London, London WC2R 2LS, UK; d.whitney@nhs.net; 3 Department of Geography and Environment at the University of Southampton, Southampton SO17 1BJ, UK; g.moon@soton.ac.uk

**Keywords:** health literacy, synthetic area estimates, general practice

## Abstract

*Background*: Low health literacy is associated with poorer health. Research has shown that predictive models of health literacy can be developed; however, key variables may be missing from systems where predictive models might be applied, such as health service data. This paper describes an approach to developing predictive health literacy models using variables common to both “source” health literacy data and “target” systems such as health services. *Methods*: A multilevel synthetic estimation was undertaken on a national (England) dataset containing health literacy, socio-demographic data and geographical (Lower Super Output Area: LSOA) indicators. Predictive models, using variables commonly present in health service data, were produced. An algorithm was written to pilot the calculations in a Family Physician Clinical System in one inner-city area. The minimum data required were age, sex and ethnicity; other missing data were imputed using model values. *Results*: There are 32,845 LSOAs in England, with a population aged 16 to 65 years of 34,329,091. The mean proportion of the national population below the health literacy threshold in LSOAs was 61.87% (SD 12.26). The algorithm was run on the 275,706 adult working-age people in Lambeth, South London. The algorithm could be calculated for 228,610 people (82.92%). When compared with people for whom there were sufficient data to calculate the risk score, people with insufficient data were more likely to be older, male, and living in a deprived area, although the strength of these associations was weak. *Conclusions*: Logistic regression using key socio-demographic data and area of residence can produce predictive models to calculate individual- and area-level risk of low health literacy, but requires high levels of ethnicity recording. While the models produced will be specific to the settings in which they are developed, it is likely that the method can be applied wherever relevant health literacy data are available. Further work is required to assess the feasibility, accuracy and acceptability of the method. If feasible, accurate and acceptable, this method could identify people requiring additional resources and support in areas such as medical practice.

## 1. Introduction

The WHO defines health literacy as “the cognitive and social skills which determine the motivation and ability of individuals to gain access to, understand and use information in ways which promote and maintain good health” [1]. Low health literacy affects a significant proportion of English adults; 6 in 10 (61%) adults aged between 16 and 65 years of age in England lack the literacy and numeracy skills to fully understand common health-related information [2].

Research has shown that, compared with people with adequate health literacy, people with low health literacy are more likely to die prematurely [3], have one or more long-term health conditions (LTHCs) [4], find any LTHCs more limiting [4], and find compliance with prescribed medication more challenging [5]. Furthermore, health literacy is also associated with lifestyle, with people with lower health literacy being more likely to exhibit unhealthy behaviours such as low levels of physical activity, poor diet/obesity, and harmful alcohol consumption [4]. Lower health literacy is also associated with lower rates of participation in disease prevention programmes such as screening, immunisation, and public health information campaigns [5].

Bodies commissioning health care in England have a legal duty to reduce health inequalities [6]. As data suggest a strong link between socio-demographic deprivation and low levels of health literacy [2], interventions focused on health literacy may have the potential to contribute to a reduction in health inequalities.

In order to understand and address the challenges of low health literacy within the health system, identification of individuals at risk of low health literacy has the potential to be useful at both population and individual patient levels. Previous work has demonstrated that statistical techniques can be applied to health literacy datasets to identify socio-demographic characteristics predictive of low health literacy in the U.S., Europe and England [7,8,9,10]. The predictive models produced from such “source” datasets, which could be described as “stage 1 models”, can only be applied in systems that contain key predictive variables, in particular education level. Education level is rarely collected in routine medical practice, precluding the use of these methods in this setting.

This project aimed to explore the accuracy and feasibility of an alternative approach, which could be described as a “stage 2” predictive model. This model used only data routinely collected in the “target” system (in this case medical care practice) and present in the “source” health literacy dataset, i.e., individual socio-demographic details and geographical area of residence. As the model included geographical area of residence, area levels of health literacy could also be estimated using this approach. We explored accuracy at both individual patient and local area level. The study was undertaken in Lambeth, an inner-city borough in London, England. Lambeth is the 22nd most deprived of the 326 boroughs in England [11], with 45% if the population identifying as being in the “non-White” ethnic group, compared to 13% in England and Wales as a whole [11,12].

## 2. Materials and Methods

Lambeth DataNet (LDN) [13] is a database of pseudonymised, patient-level, primary care data covering over 360,000 people extracted from 46 GP practices serving the inner London Borough of Lambeth. It aims to provide health care service commissioners, public health, and researchers with high quality contemporaneous primary care data to improve local services and reduce inequalities. LDN does not contain any Patient Identifiable Data (PID); post/ZIP code of residence is converted to Lower Super Output Area (LSOA) of residence and Index of Multiple Deprivation (IMD) [14]. Lower Super Output Areas are standard small-area UK census geography areas, each covering areas of residence for approximately 1600 people [15]. 

Previous work has identified the literacy and numeracy skill levels required by working-age adults in England to fully understand and use health information in common circulation [2]. Rowlands et al. describe a health literacy “competency threshold”: the literacy and numeracy skills required to fully understand and use health information in common circulation [2]; i.e., functional health literacy [16]. 

National small area estimates of health literacy were developed for LSOAs. The estimates were developed using the 2011 Skills for Life Survey (SfL) [17] and 2011 English census data [18], with embedded participant health literacy levels developed and reported previously by Rowlands et al. [2], and a multilevel synthetic estimation method widely used in past work, inter alia, on the estimation of small-area smoking prevalence [19,20,21]. Variables used in the estimates were age, sex, ethnicity, whether or not English was the first language, and deprivation of the area of residence. The synthetic estimation produced LSOA-level estimates of the proportion of the adult working-age population below the health literacy competency threshold for each LSOA in England, and a predictive model enabling the risk of an individual below the health literacy competency threshold to be determined. As the SfL survey only included working-age adults (16 years to 65 years), estimates were only calculated for this age range. The coefficients in the resulting model were converted to risk percentages for each cell in the age by sex, by ethnicity, by language preference, and by deprivation table. The intercept was the category hypothesised to have the lowest risk of having a health literacy level below the competency threshold. The Deviance Information Criterion (DIC) was calculated to assess the effectiveness of the model [22]. 

The predictive model required, at a minimum, patient age, sex and ethnicity data; other variables in the model, i.e., LSOA of residence and language preference, were not essential, but, if present, increased the accuracy of the estimation.

The algorithm used to import the risk scores derived from the predictive model into the patient records was a script file written for the R software package. The R script took a single input table, i.e., the individual patient-level data with demographic characteristics, and, after processing, returned the table in the original format with an additional column showing the health literacy risk scores. The R script also included basic data validation to check that characteristics in the input (patient) table were all available and in the expected format to prevent incorrect or unexpected results. More details of the algorithm are given in the supplementary file and Table S1.

The proportion of people below the health literacy threshold in Lambeth was compared with the proportion in the rest of England.

The validity of the model was assessed at two levels: geographical (LSOA) and individual. At LSOA level, the mean percentage risk score of adults aged 16 years to 65 years in the LSOAs according to the risk calculation using the LDN database was calculated and subtracted from the small-area estimate of risk. To eliminate boundary effects of patients living in one borough and registering with a GP practice in another borough, LSOAs were excluded where the GP registered population in the Lambeth DataNet database was >+15%/−15% of the latest ONS population estimates. To check the reliability of the algorithm coding at the individual level, a sample of 0.37% of people (*n* = 1013) was identified for accuracy estimation. There were 600 possible permutations of the demographic characteristics; however, 87 of these permutations were “empty”, i.e., with no matching patient records. One patient record was randomly selected from each of the remaining 513 permutations. A further 500 records were randomly selected from the remaining records. These 1013 records were then checked manually against lookup tables to establish the accuracy of the score.

The characteristics of individuals for whom the minimum data set for calculation of the health literacy risk score was available were compared with the characteristics of individuals for whom the risk score could not be calculated. Statistical significance was assessed using χ^2^ tests, and the strength of the association was assessed using Cramers V.

### Ethics

Lambeth DataNet is a patient-level database which does not contain personal identifiable data (PID) [13]. This study did not involve the removal of data from its agreed location (NHS Lambeth Clinical Commissioning Group (CCG)) [23], nor did it involve the access to the database by any individuals not already granted access to data for the purposes of supporting Lambeth CCG business intelligence. The project was approved by the Lambeth DataNet steering group (a body made up of local GPs, public health professionals, commissioning managers, and patient/public representation, responsible for overseeing the development and use of the database) on 22 September 2015 and the Lambeth CCG information governance steering group were informed [24]. 

As no PID were held, the project did not require NHS Ethics Approval [25].

## 3. Results

The 2011 census data for Lambeth showed 225,113 residents aged between 16 years and 65 years, of whom 156,626 (69.58%) were estimated to be below the health literacy threshold. Census data for the rest of England showed 34,329,091 residents aged between 16 years and 65 years, of whom 21,168,166 (62.07%) were estimated to be below the health literacy threshold.

On the data extraction date (31 March 2014), LDN contained the data of 364,009 people, of whom 275,706 were in the age range 16 years to 65 years. Of the 275,706 people aged 16 years to 65 years, a risk score could be calculated for 228,610 people (82.92%); in the remaining 47,096 people (17.08%), the risk score could not be calculated due to one or more missing variables essential for risk score calculation (i.e., age, sex or ethnicity). As per Lambeth CCG information Governance regulations, any patients for whom sex was not available were removed from further analyses.

### 3.1. Predictive Model

The coefficients and fit statistics for the model are shown in Table 1. The reference category was white males, aged 16 to 24 years, with English as a first language, living in a “least-deprived” area. The DIC statistic suggests that the model represents a significant improvement over a “null” model with no covariates.

### 3.2. Algorithm Accuracy

For the 109 LSOAs included in the analysis, the mean difference in the percentage health literacy risk score (i.e., the percentage of residents calculated to be below the health literacy competency threshold) arising from the application of the algorithm was subtracted from the national small area estimates. The mean difference was +1.8/−1.8 percentage points (SD 3.8). Individual-level validation using 1013 records confirmed the accuracy of the process. 

The missing data preventing the calculation of a risk score for individuals is shown in Table 2. All but 1 person had sex recorded. For the remaining 47,096 individuals with missing data, all were missing ethnicity data, of whom 88 people were also missing an IMD score due to either an un-linkable postcode (i.e., the postcode had been entered incorrectly by the General Practices or the LSOA was not in England) or was missing entirely.

Comparison of the characteristics of individuals for whom a risk score could be calculated with individuals for whom the risk score could not be calculated are shown in Table 3. This shows that individuals for whom a risk score could not be calculated due to missing data were statistically significantly more likely to be older, male, and to live in more deprived areas; however, the Cramer’s V scores were all very low, indicating that the strengths of the associations were low.

## 4. Discussion

### 4.1. Summary of Main Findings

This study shows that it is feasible to develop predictive models from “source” health literacy datasets, using variables in common with “target” systems such as health systems, and then to apply these to identify individual- and area-level risk of low health literacy. On the basis of a sample of 1013 records, a check confirmed that the algorithm was coded correctly. At LSOA area level, there was a mean difference between the register-based algorithm and a small population-based estimate of the percentage of residents below the health literacy threshold of 1.8 percentage points. This difference likely arises from three factors. Firstly, the Lambeth DataNet data were extracted in 2014, whereas the census data on which the multilevel synthetic estimates were based was collected in 2011; migration and other elements of urban change will result in differences in the population base that would be expected to increase as the temporal distance from the census date increases. Secondly, there will be differences between the resident (census) population and that registered with GPs; under-registration is a known theme in inner city areas and is likely to particularly involve groups with low health literacy. Thirdly, the multilevel synthetic estimates used data not included in the risk predictions arising from the Lambeth DataNet data, in particular language preference. Given these factors, there is a strong correspondence between the synthetic estimates and risk prediction model.

This project has shown that, in the setting used in this project, ethnicity recording is essential for the synthetic estimates to be applied. Collection and recording of patient ethnicity is now a requirement in General Practice in England, and has resulted in over 90% recording in newly registered patients [26]; however, it is not known what current levels of recording are in longer-established patients, and whether their socio-demographic characteristics differ significantly from newly registered patients. This study found that the 17.08% of the population for whom the health literacy risk score could not be calculated due to missing ethnicity data were older, male or living in more deprived areas than the rest of the population, although the strength of these associations was weak. Some members of this “missing data” population may be longer-established residents, likely to be older and living in more deprived areas, while others may be recent arrivals, more likely to be male and living in more deprived areas; all may be at significant risk of falling below threshold levels of health literacy. Consequently, it may be that people for whom the risk of poor health literacy cannot be computed should be considered at risk.

### 4.2. Strengths and Limitations of This Study

This study used high-quality robust data. The 2011 national census and national English literacy and numeracy survey data [17] were used for the synthetic estimates, and the Lambeth DataNet is a high-quality dataset extracted directly from Primary Care clinical systems [13]. The synthetic estimation techniques have been demonstrated as accurate and robust in previous studies [19,20,21].

Some limitations must, however, be acknowledged. Lambeth is an atypical borough with high levels of socio-economic deprivation, a high proportion of people from Black and Minority Ethnic groups, and lower health literacy levels when compared to the rest of England. This study requires replication in other localities to assess its wider usefulness. In addition, it must be recognised that the synthetic estimate model gives a risk score for health literacy, and not a definitive “diagnosis” for individuals. Any score applied to individuals should thus be treated as a guide only, and adjustment of communication should be made to ensure that health messages and other communications are received and understood. 

### 4.3. Implications for Practice and Research

This study highlights the importance of accurate and full collection of socio-demographic data, including postcodes, in areas of practice, such as health care, where health literacy may be key to the provision of high-quality care. While not a diagnostic tool, at an individual level, the synthetic estimates and algorithm could be used to highlight, and thus provide additional resources for, patients and clients at risk of low health literacy. At an area level, the method described here could be used to allocate staff and budgetary resources to geographical areas (such as health trusts and authorities providing social care) providing services to populations with high health literacy needs.

The models developed in this project are dependent on the datasets that form the basis of the models, i.e., the health literacy dataset and census data, combined with a knowledge of socio-demographic and geographical data present in the target data (in the case, health service data). Specifically, the “health literacy” outcome will reflect the way in which health literacy has been measured in the source dataset; in this case functional health literacy. However, the methods described here could theoretically be applied in any setting where there are baseline health literacy and census datasets, and where sufficient data are collected routinely in (for example) health care settings to enable the models to be built. Further research would need to be undertaken to assess the robustness of the resultant models in these settings.

## 5. Conclusions

This study explored the development and application of synthetic estimates for health literacy to primary care data in an inner-city area with high socio-economic deprivation and a high proportion of people from people from minority ethnic groups. If the findings are replicated in areas with different socio-demographic profiles, the methods described here may aid the delivery of resources and care to populations with low health literacy, and facilitate more health literacy research. 

## Figures and Tables

**Table 1:** Coefficient and fit statistics of predictive model.

Variable	Coefficient (Logit)	Standard Error
Intercept (white males, aged 16 to 24 years, with English as a first language, living in a “least-deprived” area)	−1.010	0.108
*Age*		
25–34	−0.196	0.103
35–44	−0.262	0.101
45–54	0.213	0.100
55–65	0.383	0.099
*Gender*		
Female	−0.147	0.057
*Ethnicity*		
South Asian	0.366	0.152
Black	0.518	0.178
Other	0.220	0.180
*First language*		
Non-English Speaker	0.876	0.125
Deprivation of area of residence		
Deprivation IMD Quartile 2	0.456	0.085
Deprivation IMD Quartile 3	0.735	0.097
Deprivation IMD Quartile 4 (most deprived)	1.244	0.090
Area-level variance	0.146	0.044
Units: Areas	828	
Units: Individuals	5815	
Estimation	MCMC	
DIC Full Model (Null Model)	7484.964 (7800.871)	
Chain Length	500,000	

IMD: Index of Multiple Deprivation; DIC: Deviance Information Criterion.

**Table 2:** Missing data preventing risk score calculation.

Missing Data Variable	*n*
missing ethnicity only	47,008
missing IMD only	0
missing ethnicity and IMD	88
missing sex	1
total missing	47,097

**Table 3:** Comparison of socio-demographic characteristics for individuals for whom a risk score could be calculated compared with individuals for whom a risk score could not be calculated.

Socio-Demographic Characteristic	People with Sufficient Data for Risk Score % (*n*)	People without Sufficient Data for Risk Score % (*n*)	χ^2^, df	*p*	Cramers V
*Total*	82.92 (228,610)	17.08 (47,097)			
*Age*					
16 years to 44 years 45 years to 65 years	83.23 (164,294) 82.12 (64,316)	16.77 (33,094) 17.88 (14,002)	48.91, 1	*p* < 0.0001	0.013
*Gender*					
Female	85.22 (117,198)	14.78 (20,328)	1025.19, 1	*p* < 0.0001	0.061
Male	80.63 (111,412)	19.37 (26,768)			
*Index of Multiple Deprivation*					
1 = most deprived 2 3 4 = least deprived	82.32 (99,138) 83.48 (101,880) 83.13 (25,780) 83.68 (1687)	17.68 (21,291) 16.52 (20,158) 16.87 (5230) 16.32 (329)	36.87, 1 *	*p* < 0.0001 *	0.015

* χ^2^ test for trend.

## Supplementary Materials

The following are available online at http://www.mdpi.com/1660-4601/15/8/1709/s1, Supplementary information and Table S1: Characteristics used to determine the health literacy score.
