# Supplementary material for: Developing and Applying Geographical Synthetic Estimates of Health Literacy in GP Clinical Systems
Source: Int J Environ Res Public Health. 2018 Aug 10;15(8):1709. doi: 10.3390/ijerph15081709 (PMC6121561; doi:10.3390/ijerph15081709)
Supplement: Supplementary file 1 [file ijerph-15-01709-s001.pdf]

Supplementary information. Algorithm details

A script file written for the R software packages was the method selected to add the risk scores to the patient data[1]. The R script was designed to take a single input table (the individual patient level data with demographic characteristics) and, after processing, return the table in the original format with two additional columns showing the risk scores for both health literacy and numeracy and literacy appended, the 5 characteristics used to determine the scores are shown in supplementary table 1.

The script used the coded information in the input file to assign a 'lookup vector' to each patient in the input table. Made up of 5 numeric characters, each representing a characteristic, this value was then used to link to the data from GM's risk score matrixes which were converted to a single flat table containing all possible permeations of the characteristics, with the 'lookup vectors', health literacy and literacy and numeracy scores
The R script was also developed to include some basic data validation to check that characteristics in the input (patient) table are all available and in the expected format to prevent incorrect or unexpected results.

---

[1] R is a software environment for statistical computing and data processing and was selected because it is free, widely available, commonly used in academic research and from 2016 will be included as part of the Microsoft SQL server package making our process easily replicable by others. The script is available from the corresponding author.

Table S1. Characteristics used to determine the health literacy score.

Characteristic	Description	
Age	As numeric vector representing age in years	
Sex	A single character vector where<br>M = Male<br>F = Female<br>U = Unknown	
Ethnicity	A numeric Coded Vector Based on ONS Ethnicity Coding	
	**White**	
	1	Welsh / English / Scottish / Northern Irish / British
	2	Irish
	3	Gypsy or Irish Traveller
	4	Any other White background
	**Mixed**	
	5	White and Black Caribbean
	6	White and Black African
	7	White and Asian
	8	Any other Mixed/Multiple ethnic background
	**Asian**	
	9	Indian
	10	Pakistani
	11	Bangladeshi
	12	Chinese
	13	Any other Asian background,
	**Black**	
	14	African
	15	Caribbean
	16	Any other Black background
	**Other Ethnic Group**	
	17	Arab
	18	Any other ethnic group
	**Not Known**	
	98	Patient refused
	99	Not Recorded
Deprivation	A numeric vector based on the IMD Quartile of the patients LSOA of residence where 1 = most deprived and 4 = least deprived	
Preferred Language	A numeric coded vector based on the patients main spoken language<br>1 = English<br>2 = any other language<br>9 = Unknown or missing	
